# Construction of an electrochemical pH sensor using one-pot synthesis of a molybdenum diselenide/nitrogen doped graphene oxide screen-printed electrode†

**DOI:** 10.1039/d4ra01708k

**Published:** 2024-05-03

**Authors:** Sujittra Poorahong, Wipawee Oin, Saowaluk Buapoon, Supinya Nijpanich, David J. Harding, Mohamed Siaj

**Affiliations:** a Functional Materials and Nanotechnology Center of Excellence, Walailak University Thasala Nakhon Si Thammarat 80160 Thailand sujittra.po@mail.wu.ac.th; b Department of Chemistry, School of Science, Walailak University Thasala Nakhon Si Thammarat 80160 Thailand; c Synchrotron Light Research Institute (Public Organization) Nakhon Ratchasima 30000 Thailand; d School of Chemistry, Institute of Science, Suranaree University of Technology Nakhon Ratchasima 30000 Thailand; e Department of Chemistry, Université du Québec à Montréal Montréal Québec H3C 3P8 Canada

## Abstract

In this study, a one-pot synthesis of a molybdenum diselenide/nitrogen-doped graphene oxide (MoSe_2_/NGO) composite was demonstrated and used for the fabrication of an electrochemical pH sensor. The MoSe_2_/NGO composite was characterized using powder X-ray diffraction, infrared spectroscopy, Raman spectroscopy, X-ray photoelectron spectroscopy, thermogravimetric analysis, scanning electron microscopy, transmission electron microscopy, energy-dispersive X-ray spectroscopy, and Brunauer–Emmett–Teller analysis. The electrochemical behavior at different pH values was determined by recording the open-circuit potential. When applied for pH detection, the MoSe_2_/NGO modified screen-printed electrode (SPE) showed good linearity with a sensitivity of 61.3 mV pH^−1^ over a wide pH range of 2–14. In addition, the pH sensor exhibited a remarkably stable response, high reproducibility, and selectivity. The sensor was used to measure the acidity or alkalinity of real food and beverage samples. The results for these samples showed a relative error of less than 10% compared with the results obtained with the commercial pH meter. The portable sensor produced by screen printing electrodes paves the way for the development of simple, cost-effective, real-time, and robust pH sensors for the pH analysis of various sample matrices for clinical diagnostics, biosensing, and cost-effective applications.

## Abstract 2

In this study, a one-pot synthesis of a molybdenum diselenide/nitrogen-doped graphene oxide (MoSe_2_/NGO) composite was demonstrated and used for the fabrication of an electrochemical pH sensor.

## Graphical abstract

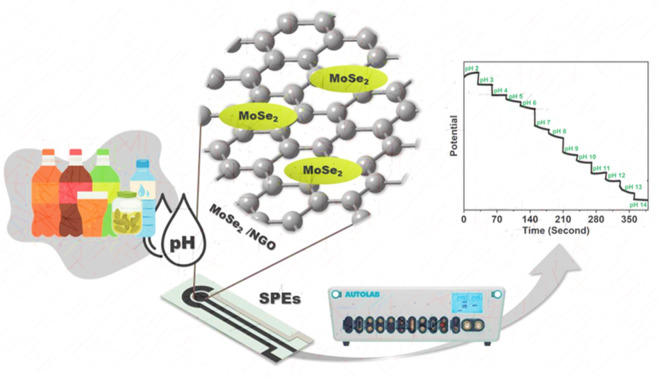

## Introduction

Measurement and pH control are among the most important parameters for various applications in industry, agriculture, biology, medicine, food, chemicals, and biological processes.^1,2^ Additionally, the pH of foods and beverages is an important quality control measure. It is essential for human health and food freshness, and is vital for food storage and transition.^3,4^ The general properties of sensing materials for food and beverage applications are good biocompatibility, and high sensitivity and anti-macrobiomolecule interference ability, and operational repeatability. Although the conventional glass pH electrode offers an accurate and stable Nernstian response over a wide pH range, it has several drawbacks, such as size constraints and mechanical fragility, which limit its application in many fields such as clinical diagnostics and environmental monitoring. In addition, the pH test strip is not sufficiently accurate and cannot applied for real-time application.

Currently, various techniques for monitoring pH, including colorimetric and electrochemical methods, such as potentiometric, amperometric, ion-selective field-effect transistor (ISFET), open circuit potential, conductometric, and optical pH sensing, have been developed to measure the pH of solutions.^1^ Among these methods, the open circuit potential (OCP) is one of the most straightforward techniques. It measures the different potentials between the working electrode depending on the charges present in solution medium.^5^ Many types of electrochemical pH sensors have been developed using various types of materials, such as WO_3_, IrO_2_, RuO_2_, SnO_2_, and Co_3_O_4_, which can be easily produced into nanostructures with chemical stability, low cost, biocompatibility, catalytic activity, and high sensor performance for various applications.^6–8^ However, constant optimization and calibration were required because of their operational instability. Other types of sensors are polymer-based, biological pH sensors that require complicated instruments and often have a limited pH detection range or temperature requirements.^6–8^ Therefore, portable pH sensors with wide detection ranges and long-term stability are required.

Graphene-based materials, especially graphene oxide (GO), are widely used in physical and chemical research owing to their high surface area, thermal conductivity, and electron mobility compared to typical semiconductor materials.^9,10^ GO can be used in electrochemical pH sensors because its surface contains various functional groups such as carboxylic acids, phenols, quinones, and carbonyl groups, which are sensitive to pH and undergo protonation and deprotonation depending on the pH of the solution.^1^ However, in practical applications, severe agglomeration and re-stacking by van der Waals interactions leads to a decrease in properties such as the specific surface area, diffusion rate, and electrical conductivity.^11^ To prevent such agglomeration, nitrogen-doped GO (NGO) has been shown to be an easy method to restore many advantageous properties of GO.^11,12^ Additionally, molybdenum diselenide (MoSe_2_), a transition metal dichalcogenide (TMD), is a low-cost semiconductor with high chemical stability and excellent thermal and electrical conductivities.^13,14^ This material is widely used in electrochemical sensors and may improve the performance of GO materials.^13^

In this study, a one-pot fabrication of an inexpensive molybdenum diselenide and NGO composite-modified screen-printed carbon electrode (MoSe_2_/NGO/SPE) was used as an electrochemical pH sensor. The modified electrodes were performed in buffer solutions of pH 2–14 by recording the OCP values. The hybrid MoSe_2_/NGO/SPE electrodes showed a high electrochemical active surface area and high specific capacitance, which significantly improved good Nernstian response. The electrochemical pH sensor also exhibited excellent reproducibility, and high selectivity against other possible interfering ions. Finally, the pH of real food and beverage samples, such as lime juice, cola, green tea, pickled vegetable, drinking water, alkali water, and vinegar was successfully determined, and the sensor showed repeatability, stability, and comparable results to which obtained from the commercial pH meter. Hence, the proposed sensor has high potential for application in the food and beverage industry for real-time continuous monitoring of pH in production processes.

## Experimental

### Preparation of composite MoSe_2_ on NGO

The GO and NGO synthesis procedures are described in the ESI.† The composite MoSe_2_ on NGO was synthesized using a solvothermal method.^14^ Firstly, 0.2 g of NGO was dispersed in 20 mL of DI water and sonicated for 1 h. Then 0.42 g of ammonium molybdate tetrahydrate and 1.065 g of selenium dioxide were added to the mixture and stirred for 45 min. Subsequently, 50 mL ethylenediamine was added while stirring continuously for 15 min. The final mixture was transferred to a 100 mL Teflon container with stainless-steel autoclave and heated at 200 °C for 24 h. The final product was washed several times with DI water/ethanol and dried.

### Fabrication of the electrochemical pH sensor

The electrochemical pH sensor was prepared by dispersing 3 mg of MoSe_2_/NGO in 1.0 mL of DI water–ethanol (4 : 1) solution and 30 μL of Nafion, followed by sonication for at least 30 min to form a homogeneous suspension. Then, 2.0 μL of the 3 mg mL^−1^ slurry was drop-cast onto the working electrode of the screen-printed electrode (SPE). The electrode assembly comprised carbon as the working and counter electrode, and Ag/AgCl as the reference electrode. Finally, the electrodes were dried at room temperature. To compare the detection performances, SPEs modified with NGO and MoSe_2_ were also prepared by the same procedures.

### The electrochemical pH measurement

The unmodified SPE, and modified SPE with NGO, MoSe_2_ and MoSe_2_/NGO were used to measure the pH of the buffer solution by monitoring the OCP values *versus* a Ag/AgCl reference electrode. Working solutions were prepared using buffer solutions (4, 7, and 10) and adjusted with NaOH or HCl solutions to obtain pH values ranging from 2 to 14. The measurements were carried out under ambient atmosphere and at room temperature.

### Real samples analysis

The MoSe_2_/NGO modified screen-printed electrode was used to detect pH of various real samples including drinking water, cola, alkali water, vinegar, lime juice, and pickled vegetable without any sample preparation step. Real sample results were validated using a commercial pH meter.

## Results and discussion

### Morphology and structure of the synthesis materials

**Fig. 1:**
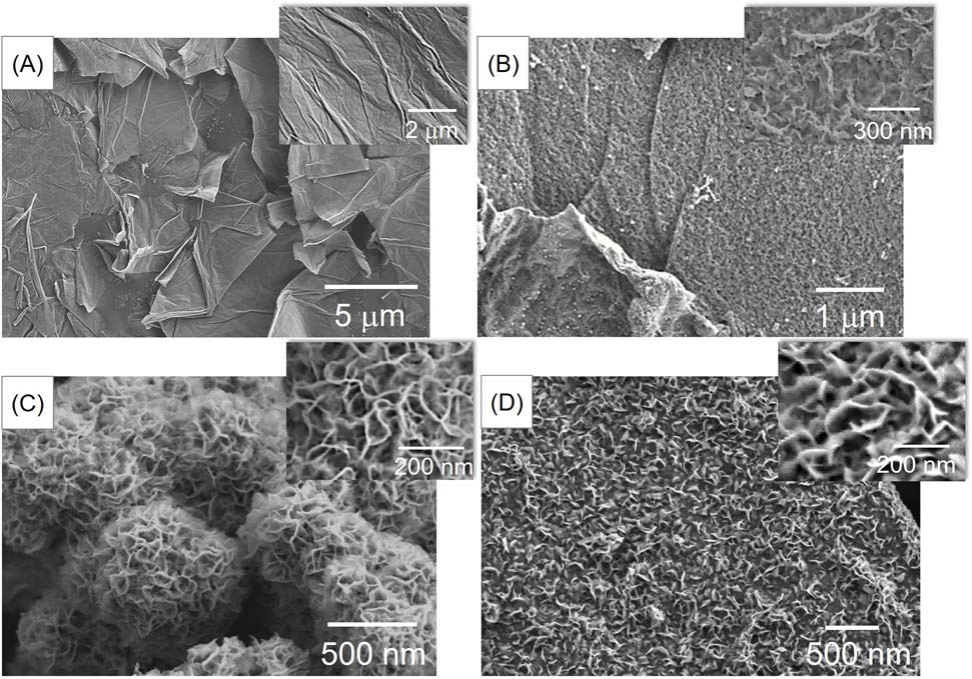
SEM images of GO (A), NGO (B), pure MoSe_2_ (C), and MoSe_2_/NGO (D).

The morphology and elemental composition of the materials were investigated using field-emission scanning electron microscopy (FE-SEM) and energy-dispersive X-ray spectroscopy (EDS). Fig. 1(A) shows that GO consists of large and smooth sheets. After doping nitrogen into the GO (Fig. 1(B)), the smooth surface became rough. Additionally, Fig. 1(C) shows that pure MoSe_2_ exhibits a rose-like microsphere morphology with numerous petals. The incorporation of MoSe_2_ into the material (Fig. 1(D)) leads to the growth of petals on the NGO surface, which may increase the surface-active area and electrical conductivity, thereby enhancing the effectiveness of the pH measurement. In addition, the elemental composition of MoSe_2_/NGO was verified by EDS spectra as shown in Fig. S1† which consists of C, N, O, Mo, and Se elements which matched the NGO and MoSe_2_ structure. The EDS mapping profiles were further examined and have revealed the presence of Mo, Se, C, N, and O elements, which are distributed uniformly across the sample as shown in Fig. S2.†

**Fig. 2:**
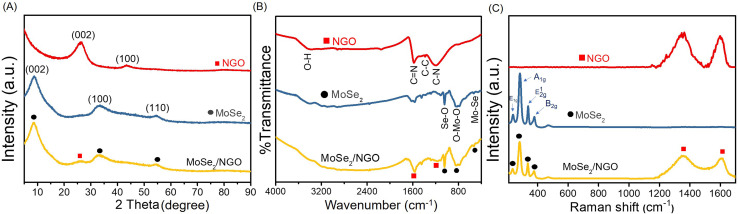
The PXRD patterns (A), FTIR spectra (B) and Raman spectra (C) of NGO, MoSe_2_ and MoSe_2_/NGO materials.

The phase identification of the synthesized NGO, MoSe_2_, and MoSe_2_/NGO was performed using powder X-ray diffraction (PXRD), and the patterns are shown in Fig. 2(A). The characteristic peaks of NGO showed two peaks at 2*θ* approximately 26.3° and 43.4°, corresponding to the (100) and (002) diffraction planes, respectively, which is consistent with the NGO structure.^11^ The peaks at 2*θ* around 9.0°, 33.0°, and 54.3° correspond to the (002), (100), and (110) diffraction planes, respectively, which can be assigned to the hexagonal 2H–MoSe_2_ phase (JCPDS 29-0914).^13,15–17^ The distance between each plane (Table S1†) was calculated using Bragg's law, 2*d* sin *θ* = *nλ*, where *d* is the distance between planes, *θ* is the Bragg angle, *λ* is the wavelength of the incident radiation (1.5406 Å), and *n* is the degree of reflection.^18^ The diffraction patterns of MoSe_2_/NGO sample confirm the presence of NGO and MoSe_2_ in its structure. Additionally, it can be observed that the peaks at 2*θ* around 25° for MoSe_2_/NGO have a low intensity. This may be due to the growth of MoSe_2_ on the NGO surface, which overshadowed the NGO signal.

The functional groups of all materials were measured by FT-IR spectroscopy, as shown in Fig. 2(B). The FTIR spectrum of GO presented a peak at 3420 cm^−1^, which was attributed to the stretching vibration of O–H. The absorption peaks at 2920 and 2852 cm^−1^ are assigned to C–H stretching. The peaks located at 1724, 1627, 1384, and 1072 cm^−1^ correspond to C=O stretching, C=C vibrations of the unoxidized graphitic domains, and C–O–C and C–OH stretching, respectively.^19^ The spectrum of NGO showed peaks at 1519 and 1213 cm^−1^, which were assigned to the C=N and C–N stretching modes of NGO, respectively.^20^ The transmittance peaks of MoSe_2_ at 461 cm^−1^ belong to the vibration of the Mo–Se bond, and those at 1045 and 829 cm^−1^ correspond to the Se–O and O–Mo–O bonds, respectively, which can be formed when exposed to air.^17,21^ The FTIR spectrum of MoSe_2_/NGO contained these functional groups, indicating the presence of NGO and MoSe_2_ in the composite material.

The Raman spectra of the samples are shown in Fig. 2(C). In the spectrum of MoSe_2_/NGO (yellow line), six main peaks were observed at 195, 235, 281, 336, 1344, and 1604 cm^−1^, which align with the characteristic peaks of MoSe_2_ (blue line) and NGO (red line) structures. The peaks at 235 and 281 cm^−1^ are related to the out-of-plane *A*_1g_ and in-plane *E*_2g_^1^ vibrational modes of MoSe_2_, respectively.^22^ The peak at 195 cm^−1^ is attributed to the *E*_1g_ mode, which is forbidden in backscattering but appears due to the resonance effect. The peak at 336 cm^−1^ is connected to the 2-phonon frequency of the *B*_2g_ branch at the M point.^23^ The magnification of the MoSe_2_ spectrum is depicted in Fig. S3.† The peaks at 1344 cm^−1^ (D band) and 1604 cm^−1^ (G band) were assigned to the defects and disorder of the graphene structure, and *E*_2g_ bond stretching of the sp^2^ carbon atoms of the NGO structure, respectively.^12^

**Fig. 3:**
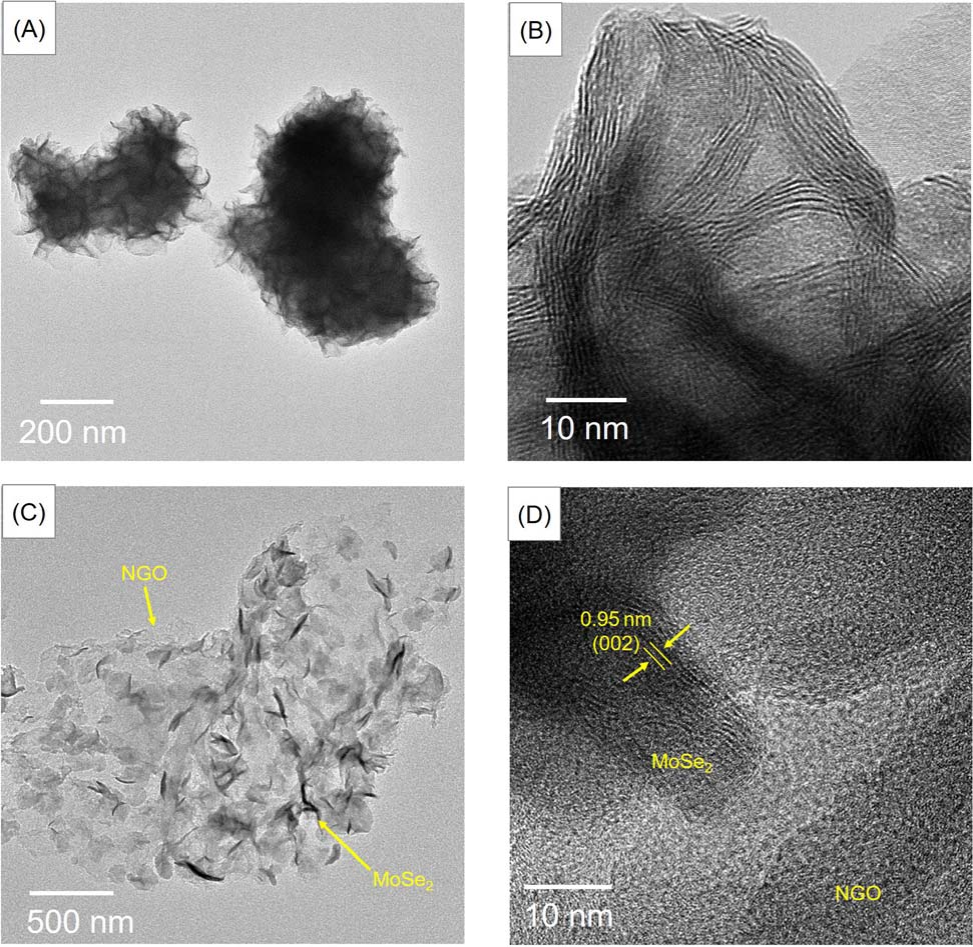
TEM images of MoSe_2_ (A and B) and MoSe_2_/NGO (C and D).

TEM images of the as-synthesized materials are shown in Fig. 3. The MoSe_2_ image in Fig. 3(A and B) shows a bulk microsphere covered with petals. Furthermore, from Fig. 3(C and D), the image of MoSe_2_/NGO clearly reveals two phases, with the MoSe_2_ phase present as dense petals, while the NGO exists as transparent sheets. The lattice fringe patterns reveal a *d*-spacing of 0.95 nm for MoSe_2_/NGO (Fig. 3(D)), corresponding to the (002) plane of MoSe_2_, closely matching the PXRD result.

**Fig. 4:**
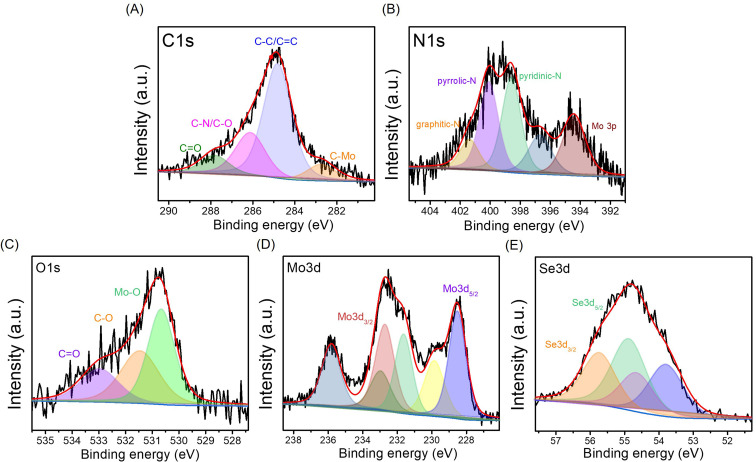
High-resolution XPS spectra of MoSe_2_/NGO: C1s (A), N1s (B), O1s (C), Mo3d (D), and Se3d (E).

The surface chemistry of MoSe_2_/NGO was analyzed by X-ray photoelectron spectroscopy (XPS). The survey spectra of MoSe_2_/NGO and NGO are shown in Fig. S4(A).† The survey spectra indicated the presence of Mo, Se, C, N, and O in MoSe_2_/NGO. In contrast, only C, N, and O were identified in NGO in the binding energy range. The high-resolution spectra of the C1s, N1s, O1s, Mo3p, and Se3p regions are shown in Fig. 4. The XPS spectrum in the region of the C1s peak, as shown in Fig. 4(A), consists of four peaks with binding energies at 287.8, 286.1, 284.8 and 282.7 eV assigned to C=O, C–N/C–O, C–C/C=C, and Se LMM Auger lines, respectively.^21,24^ The XPS spectrum in the region of the N1s peak as shown in Fig. 4(B), is composed of five main peaks. The peaks with binding energies of 401.4, 400.1 and 398.6 eV corresponded to graphitic-N, pyrrolic-N and pyridinic-N, respectively,^25,26^ while the peaks with binding energies of 394.4 and 396.7 eV were attributed to Mo3p peaks. When compared with the spectra of NGO (Fig. S4(B and C)†), the C1s and N1s peaks of MoSe_2_/NGO had functional groups similar to those of NGO, confirming the existence of the NGO structure. The O1s peak, as shown in Fig. 4(C), consists of three peaks with binding energies of 533.0, 531.5, and 530.7 eV assigned to C=O, C–O, and Mo–O bonds, respectively.^17,26^ The Mo3d (Fig. 4(D)) doublet peaks with binding energies of 231.6 and 228.5 eV were assigned to Mo3d_3/2_ and Mo3d_5/2_ of MoSe_2_, respectively. These peaks were attributed to the +4 oxidation state of Mo.^27^ In addition, the Mo3d_3/2_ and Mo3d_5/2_ peaks with binding energies of 232.7 and 229.5 eV, respectively, were attributed to MoO_2_, and Mo3d_3/2_ and Mo3d_5/2_ peaks with binding energies of 235.8 and 232.9 eV, respectively, corresponding to MoO_3_, which may be formed by surface oxidation to form the Mo^6+^ oxidation state.^26,27^ MoSe_2_ is known to form a native oxide when exposed to air.^28^Fig. 4(E) shows the XPS spectrum in the region of the Se3d peak, which can be fitted into four peaks. The Se3d doublet peaks with binding energies at 55.7 eV and 54.9 eV were assigned to Se3d_3/2_ and Se3d_5/2_ of Se(0) (elemental selenium), respectively.^17^ The Se3d doublet peaks with binding energies at 54.6 eV and 53.8 eV corresponded to Se3d_3/2_ and Se3d_5/2_ characteristic of Se^2−^ representing the metal–selenide bonds of MoSe_2_.^17^ The Mo3d (Fig. S4(D)†) and Se3d (Fig. S4(E)†) peaks of MoSe_2_/NGO were not significantly different from those of MoSe_2_, suggesting that the growth mechanisms were similar. The XPS results confirmed that MoSe_2_/NGO was successfully synthesized, consisting of MoSe_2_ and NGO structures.

The specific surface areas and average pore diameters of the samples were monitored using BET surface area analysis, as shown in Table S2.† It can be observed that doping nitrogen into GO resulted in a higher specific surface area (436.86 m^2^ g^−1^) than pure GO (297.12 m^2^ g^−1^) because heteroatom doping can prevent aggregation or re-stacking, leading to an increased specific surface area.^11,12^ The incorporation of NGO into the MoSe_2_ material enhanced the specific surface area of MoSe_2_, which corresponded to the FE-SEM results, and may increase the sensitivity of pH detection.^29^ Fig. S5† presents the nitrogen adsorption–desorption isotherm of the MoSe_2_, NGO, and MoSe_2_/NGO samples. The nitrogen adsorption–desorption experiment revealed a Type IV isotherm for all samples, indicating a mesoporous structure with a characteristic H4 hysteresis loop. The presence of H4 hysteresis, commonly observed in micro-mesoporous material structures, suggests a diverse range of pore shapes.

The thermal stability of the materials was measured by TGA and ΔTG under N_2_ gas as shown in Fig. S6.† The results showed that the decomposition temperatures of MoSe_2_/NGO between 250–350 °C (∼6 wt%) and 350–500 °C (∼54.8 wt%) probably originate from the loss of intercalated water molecules and MoSe_2_, respectively. This was similar to the TGA curve of MoSe_2_, indicating the presence of MoSe_2_ in the structure.^30^ The content of MoSe_2_ nanosheets on the NGO is found by thermogravimetric analysis to be 54.8 wt%.

### Electrochemical pH sensing of the materials

#### Open circuit potential values of the solution with different pH

**Fig. 5:**
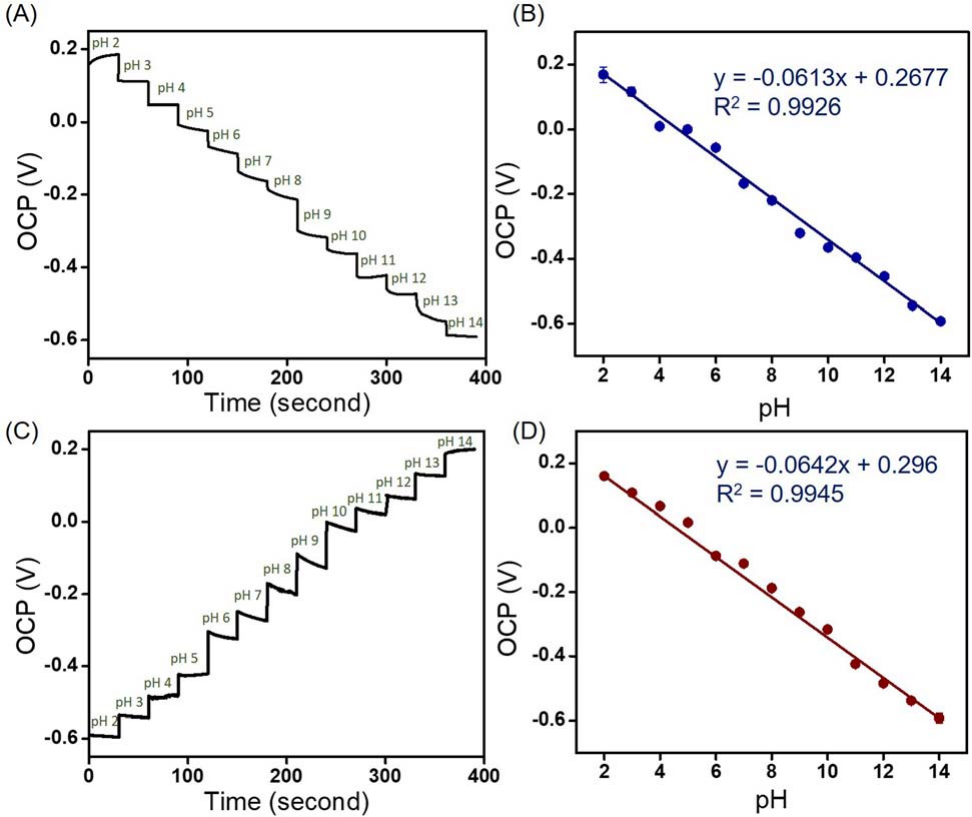
Open-circuit potential responses of pH 2–14 solutions (A), the corresponding calibration curves (B), open-circuit potential responses of pH 14–2 solutions (C), and the corresponding calibration curves (D) using MoSe_2_/NGO/SPE.

The open circuit potential (OCP) was used to illustrate the electrochemical performance of the MoSe_2_/NGO pH sensor under ambient condition. OCP is the detection of the resting potential measured between the reference and working electrodes. The counter electrode (necessary to pass current through the cell) circuitry of the potentiostat is bypassed. The OCP was evaluated over a period of 30 seconds for each pH value, and the average OCP was then collected. To ensure accuracy, the experiments were conducted three times independently. Subsequently, calibration curves and error bars were plotted using the average OCP from the three independent experiments. The OCP values *versus* time revealed that the responses decreased as the pH increased from 2 to 14, as shown in Fig. 5(A). The relationship between the OCPs and pH showed a significant linear fit (*R*^2^ = 0.9926), and the function can be expressed as *y* = −0.0613*x* + 0.2677. Thus, the sensitivity can be defined as 61.3 mV pH^−1^ in the pH range of 2–14 (Fig. 5 (B)). Subsequently, the OCP values were investigated from base to acid in the pH range 14–2 (Fig. 5(C)), and the sensitivity was found to be 64.2 mV pH^−1^. The sensitivity obtained from the acid-to-base direction and *vice versa* is comparable to the theoretical sensitivity of 59 mV pH^−1^ based on the Nernst equation, which means that almost one electron per H^+^ ion is transferred during the redox reaction.^31^ In the MoSe_2_/NGO sensor, polar functional groups, including –OH, –COOH, C=N, and C–N are presented on the surface of the materials, as shown in the FT-IR spectrum. Under acidic conditions, hydrogen ions (H^+^) attach to the inner Helmholtz plane, generating a positive charge in the material. However, when exposed to an alkali electrolyte, hydroxyl (OH^−^) is adsorbed on the inner Helmholtz plane, resulting in a negative charge.^32^ In addition, the large number of MoSe_2_ nanosheets on the surface of NGO, contributed to the excellent adsorption of H^+^ or OH^−^. Both H^+^ and OH^−^ ions are non-faradaic (capacitive), leading to the formation of supercapacitors or electrochemical capacitors at the electrode/electrolyte interface. Therefore, the OCP value is proportional to the pH of the solution. For comparison purposes, the OCPs were investigated in acid to base and *vice versa* using NGO/SPE and MoSe_2_/SPE; the corresponding relationship between the OCP and pH is shown in Fig. S7.† As summarized in Table S3,† NGO/SPE and MoSe_2_/SPE exhibited sensitivity values of 42.2 and 61.7 mV pH^−1^, respectively from pH 2–14 at room temperature. It can be observed that the composite MoSe_2_/NGO/SPE provided sensitivity values closer to the Nernstian response value than the modified electrodes and *R*^2^ values close to 1. However, different sensitivity of the proposed composite material under acid-to-base and base-to-acid conditions were still observed. But the different sensitivity was only 2.9 mV which was the lowest value among the modified sensors. The difference in sensitivity may be due to many reasons such as the repeated use of an electrode in the same pH buffer, leading to random output voltages,^33^ slow response behavior over the full pH range,^34^ and faster diffusion of H^+^ ions to the buried site than that of OH^−^ ions because of the different sizes of H^+^ and OH^−^ ions.^34^ Moreover, the presence of MoSe_2_ on the nanosheets on the surface of NGO reduces the hysteresis typically found when measuring from low to high pH or *vice versa*. This indicates that the MoSe_2_ surface is strongly physically adsorbed, which facilitates the rapid and complete recovery of the material's surface under various pH conditions.^35^ These observations suggest that the high specific surface area and distribution of MoSe_2_ on the NGO surface can improve electrical conductivity, sensitivity, and reproducibility. The performance parameters of the proposed pH sensing material have been compared to other reported methods bases on the OCP determination in Table 1. The results indicate that the proposed sensor exhibits a wide pH range and superior sensitivity compared to previous methods.

**Table 1:** Structures and sensing performance of different pH sensors

Materials	Linear pH range	Sensitivity (mV pH^−1^)	Ref.
MoSe_2_/NGO on screen-printed carbon electrode	2–14	61.3	This work
Hydrothermally reduced graphene oxide	2–12	52	1
Polyaniline@oily polyurethane/polypropylene spunbonded nonwoven fabric	2–8	67.67	36
RuO_2_ on screen-printed carbon electrode	3–13	51.17	37
Niobium electrode	2–12	41	38
ITO-rGO/PANI	2–8	62.3	39
Pigment melanin on screen-printed carbon electrode	5–8	62	40
MnO_2_ on graphenized pencil lead electrode	1.5–12.5	57.051	41

To investigate the surface area and electrical conductivity of the MoSe_2_/NGO/SPE, the cyclic voltammetry and electrochemical impedance spectroscopy with [Fe(CN)_6_]^3−^/[Fe(CN)_6_]^4−^ as the redox couple were employed. For active surface area, the electrochemical responses of these different electrodes which were measured at a variety of scan rates (10–200 mV s^−1^) and can be seen in Fig. S8.† The curve was then plotted between *I*_pa_ and the square root of the scan rate (*v*^1/2^) as shown in ESI Data Fig. S9.† Thus, the effective surface area was calculated using the Randles–Sevcik equation. After modifying the SPE (5.02 cm^2^) surface with NGO (9.38 cm^2^) and MoSe_2_ (11.7 cm^2^), the surface area increased 1.9 and 2.3 times, respectively. When hybrid NGO/MoSe_2_ were used to modify the SPE surface, the resulting material (14.0 cm^2^) exhibited the largest effective surface area in this series. The electron transfer resistance between the solution and the electrode surface (*R*_et_) was further investigated. The Nyquist plots (Zim *vs.* Zre) are shown in Fig. S10.† The resistance of the electron transfer between the solution and the electrode surface (*R*_et_), the resistance of the solution (*R*_s_), the constant phase element (CPE) and the Warburg element (*Z*_W_) of the Randles equivalent circuit model (inset) were obtained from the fitted data of these Nyquist plots. The *R*_et_ of NGO/SPE and MoSe_2_/SPE was 3350 Ω and 2250 Ω, respectively. Modification of the SPE surface with hybrid NGO and MoSe_2_ led to a decrease in *R*_et_ to 915 Ω, indicating an improvement in conductivity and electron transfer efficiency.

#### Reproducibility, stability, selectivity, and real-time measurement

**Fig. 6:**
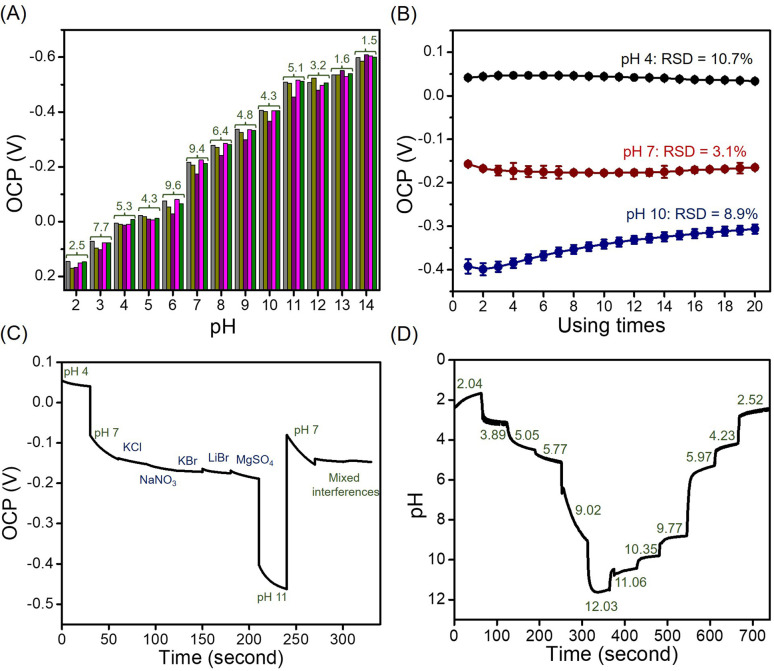
OCP values at pH 2–14 using five different MoSe_2_/NGO/SPE sensors (green labeled: % RSD of five sensors) (A), OCP values in the buffer solution at pH 4, 7, and 10 were determined at various measurement times using a single MoSe_2_/NGO/SPE (B), real-time measurements in the present of interfering species (C), and real-time measurements using MoSe_2_/NGO/SPE and pH values using a pH meter (labeled in green) (D).

Reproducibility was tested by fabricating five of the MoSe_2_/NGO modified SPE sensors and examining the OCP in buffer solutions (pH 2 to 14). The results are shown in Fig. 6(A). The calculated percentage relative standard deviations in the measured pH range were 1.5–9.6%.

To confirm the difference between the electrode preparations, the OCP for each pH value obtained from the five sensors was calculated using two-way analysis of variance (ANOVA). The results showed no significant differences, indicating that all electrode preparations had good reproducibility and homogeneity (*p* > 0.05).

The stability of the MoSe_2_/NGO modified SPE was investigated by repeated 20 measurements of the OCP in pH buffer solutions (pH 4, 7, and 10) (Fig. 6(B)). The OCP values were maintained at over 90% after 20 detection cycles, suggesting high stability of the sensors in acidic, neutral, and basic media. Moreover, stable OCP values in acidic and neutral media were established almost immediately after the second measurement, which may be because of the stability of some functional groups in the material.^1^ In alkaline solutions, a higher deviation of the open-circuit potential was observed, which indicates slow diffusion of OH^−^ for adsorption and desorption on the surface materials, and possible competitive adsorption may occur.

To evaluate the selectivity of the sensor, some possible interfering substances, *i.e.,* NaNO_3_, KCl, KBr, LiBr, and MgSO_4_, which are normally found in real samples, were analyzed using the proposed sensor. The experiment was performed three times with the average OCP response shown in Fig. 6(C). When measured in a pH 7 solution containing 1.0 M of each interference species and 1.0 M of mixed interference species, there was a negligible change in the OCP values. Hence, it can be concluded that our sensor has a high selectivity for other cations and anions.

Furthermore, real-time measurements of the sensor using the open-circuit potential while continuously changing the pH of the solution by adding HCl and NaOH are shown in Fig. 6(C). The OCP changed upon adjusting the pH of the buffer solution every minute, with a response time of less than a minute, except at pH 7–10, where a slower response was observed. This was due to the slow diffusion of the surface materials in the alkaline range, which was also observed in the stability test. Simultaneously, the accuracy was tested by comparing the values obtained from the pH meter (labeled in green). The results showed no significant differences between the methods using two-way ANOVA (*p* > 0.05).

### Real food samples analysis

**Fig. 7:**
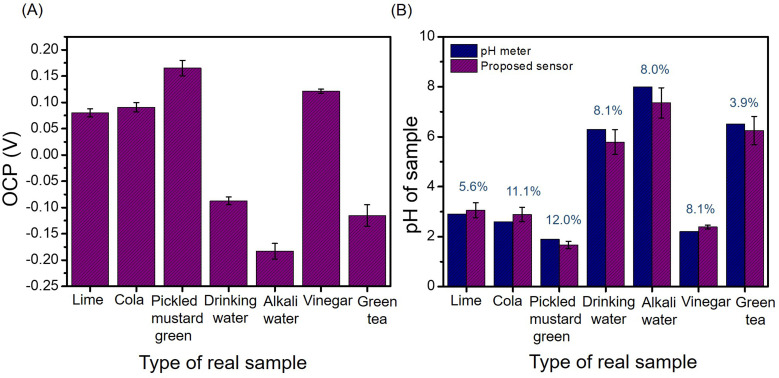
OCP values of real samples over MoSe_2_/NGO/SPE (A) and the corresponding pH values obtained from the MoSe_2_/NGO/SPE sensor and commercial pH meter (indicated in blue: % difference in pH values between the proposed sensor and commercial pH meter) (B).

To ascertain the potential application for practical sample analysis, the proposed sensor was used to measure the acidity or alkalinity of food and beverage samples, such as lime juice, cola, green tea, pickled vegetable, drinking water, alkali water, and vinegar. As shown in Fig. 7(A), the OCP values of each sample were recorded three times. The RSDs of the measurements ranged from 3–10% exhibited good stability and repeatability in real samples. The OCP values were converted to pH using a calibration curve and compared with the pH values recorded using a commercial pH meter, the results are shown in Fig. 7(B). The relative errors were less than 12% (blue label). In addition, the pH values obtained using the two methods were statistically compared using a *t*-test. The results showed no statistically significant differences between the methods (*p* > 0.05). That is, this newly proposed sensor for measuring the pH of a solution is effective and feasible. Portable sensors produced using screen printing electrodes pave the way for the development of simple, cost-effective, real-time, and robust pH sensors for the pH analysis of various sample matrices for clinical diagnostics, biosensing, and environmental monitoring applications.

## Conclusions

MoSe_2_/NGO was successfully synthesized using a facile one-pot solvothermal method. The material was then drop-cast onto a screen-printed electrode (SPE) and used as an electrochemical pH sensor *via* the open-circuit potential. The sensitivity of the MoSe_2_/NGO/SPE was 61.3 mV pH^−1^ (*R*^2^ of 0.9926) for pH 2–14. The sensor had a short response time and Nernstian response that was better than pure MoSe_2_ and pure NGO indicating that the distribution of MoSe_2_ on NGO surface can improve conductivity, decrease hysteresis loops, and increase the surface area of the composite permitting a wide detection range, high conductivity, and sensitivity. Moreover, MoSe_2_/NGO/SPE showed high repeatability, high stability, and good performance for real-time detection of pH changes. In addition, when the composite material was used as a pH sensor in real food and beverages, it exhibited a relative error of less than 12% compared with a commercial pH meter and maintained high stability, although the real samples presented a variety of interferences. Based on these features and advantages, this effective pH sensor can be used in various applications, such as in the food, beverage, and environmental industries.

## Author contributions

SP: conceptualization; data curation; formal analysis; funding acquisition; investigation; methodology; project administration; resources; supervision; validation; and writing – review & editing. WO: conceptualization; data curation; formal analysis; methodology; visualization; roles/writing – original draft; and writing – review & editing. SB: data curation; methodology; validation; review & editing. SN: data curation; formal analysis; investigation. MS: conceptualization; validation; and writing – review & editing. DJH: validation; supervision; and writing – review & editing.

## Conflicts of interest

There are no conflicts to declare.

## Supplementary Material

RA-014-D4RA01708K-s001
