# Supplementary material for: Construction of an electrochemical pH sensor using one-pot synthesis of a molybdenum diselenide/nitrogen doped graphene oxide screen-printed electrode
Source: RSC Adv. 2024 May 3;14(21):14616–23. doi: 10.1039/d4ra01708k (PMC11066617; doi:10.1039/d4ra01708k)
Supplement: RA-014-D4RA01708K-s001 [file RA-014-D4RA01708K-s001.pdf]

Electronic Supplementary Material (ESI) for RSC Advances.
This journal is © The Royal Society of Chemistry 2024

Supplementary Data

Construction of an electrochemical pH sensor using one-pot synthesis of a molybdenum diselenide/nitrogen doped graphene oxide screen-printed electrode

Sujittra Poorahong[a,b,*], Wipawee Oin[a], Saowaluk Buapoon[a], Supinya Nijpanich[c], David J. Harding[a,d], Mohamed Siaj[e]

[a]Functional Materials and Nanotechnology Center of Excellence, Walailak University, Thasala, Nakhon Si Thammarat 80160, Thailand

[b]Department of Chemistry, School of Science, Walailak University, Thasala, Nakhon Si Thammarat 80160, Thailand

[c]Synchrotron Light Research Institute (Public Organization), Nakhon Ratchasima 30000, Thailand

[d]School of Chemistry, Institute of Science, Suranaree University of Technology, Nakhon Ratchasima, 30000, Thailand

[e]Department of Chemistry, Université du Québec à Montréal, Montréal, Québec H3C 3P8, Canada

*Corresponding author; E-mail: sujittra.po@mail.wu.ac.th

Materials and chemicals

Graphite powder (~325mesh, 99.8% purity) was from Alfa Aesar, USA. Ammonium molybdate tetrahydrate and potassium permanganate were from KEMAUS, Australia. Sulfuric acid phosphoric acid and hydrochloric acid ethanol were form QЁREC, New Zealand. Selenium dioxide, Urea and Nafion-117 were obtained from Sigma-Aldrich, USA. Screen-printed electrode, SPE (BioDevice Technology, Ltd. Japan). All other chemicals and reagents were analytical grade.

Preparation of graphene oxide (GO)

Graphene oxide was prepared using a modified Hummers' method, as previously reported [1]. First, 3 g of graphite powder, 360 mL of sulfuric acid, and 40 mL of phosphoric acid were added to a 1 L flask. The mixture was then stirred for 30 min at 55-60 °C. $KMnO_4$ (18 g) was then slowly added to the suspension. After that, the mixture was continuously alternated between stirring for 30 min and ultrasonication for 30 min, 6 times, at 55-60 °C. After stirring, 30% H_2O_2 (10 mL) was added to the suspension, 600 mL of DI water at 0 °C and the mixture was stirred overnight. Hydrochloric acid (100 mL) and ethanol (100 mL) were added to the resulting suspension. Finally, the suspension was washed several times with DI water and dried.

Synthesis of nitrogen doped graphene oxide (NGO)

The as-prepared GO (40 mg) was dispersed in 20 mL of DI water and the suspension was sonicated for 1 h at room temperature. Subsequently, 12 g of urea was added to the suspension and sonicated for 2 h. The suspension was transferred and sealed in a 100 mL Teflon-lined stainless-steel autoclave and heated at 170 °C for 24 h. The material was then frozen at-80 °C overnight and freeze-dried. The product was calcined at 600 °C for 1 h at a heating rate of 10 °C min^{-1} under Ar atmosphere. The final product was washed several times with DI water/ethanol and dried.

Characterization

The powder X-ray diffraction (XRD) data was collected on an X-ray diffractometer (Empyrean, PANalytical, Netherlands) with a Cu X-ray tube, X-ray generator: 40 kV & 30 mA, Wavelength: 0.154 nm (CuKα), Scan range: (2Θ): 0.026°, Time/step: 70.125 sec. The FTIR spectra were recorded as KBr discs on a Bruker Tensor 27 FT-IR spectrometer using OPUS software in the range of 400-4000 cm^{-1}. The Raman data were collected with a laser: 532 nm excitation a spectral range of 3500–50 cm^{-1} on a Thermo Fisher Scientific, DXR SmartRaman. Field emission scanning electron microscopy (FESEM) and energy-dispersive X-ray spectroscopy (EDS) were performed on a Merlin compact (Zeiss) with an accelerating voltage of 5 kV and a working distance of 8.4 mm using the secondary electron imaging mode. Field-Emission Transmission Electron Microscopy (FE-TEM) was performed on a Thermo Scientific Talos F200i. N_2 physisorption was measured using the static volumetric N_2 gas adsorption method with a surface area and porosity analyzer (BET) (ASAP2460, Micromeritics, USA) to obtain the Brunauer-Emmett-Teller (BET) surface area and pore size distribution. The composites were analyzed using a thermogravimetric analyzer (Pyris 1 TGA, PerkinElmer, USA) from 30 °C to 1000 °C at a ramp rate of 5 °C/min under N_2 flow.

X-ray photoelectron spectroscopy (XPS) experiments were performed using a PHI5000 VersaProbe II (ULVAC-PHI, Japan) equipped with a hemispherical spherical analyzer. The excitation source was monochromatic Al Kα radiation (1486.6 eV), operated at 25 Watts. The sample charging during the measurement was compensated for by an electron flood gun. All spectra were carefully analyzed using the Multipak software. The binding energy scale was calibrated from adventitious carbon using the C1s peak at 284.8 eV. A Shirley-type function was used for the background subtraction. The constraint due to multiple spin-orbit peaks was applied. The FWHM, peak location, and optimal mix of the Lorentzian and Gaussian functions were determined to obtain the best fit.

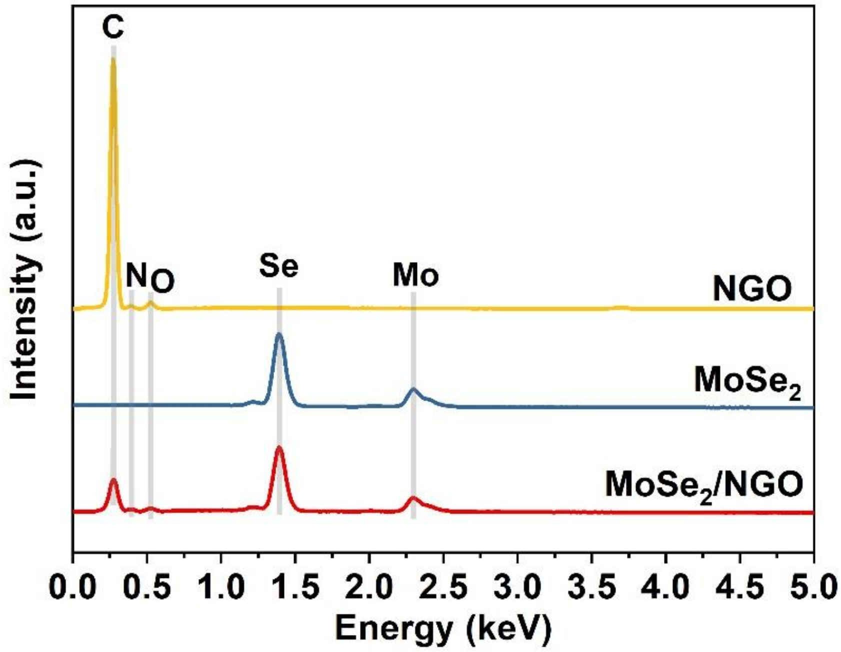

Figure S1 EDS spectra of NGO, pure $MoSe_2$, and $MoSe_2$/NGO.

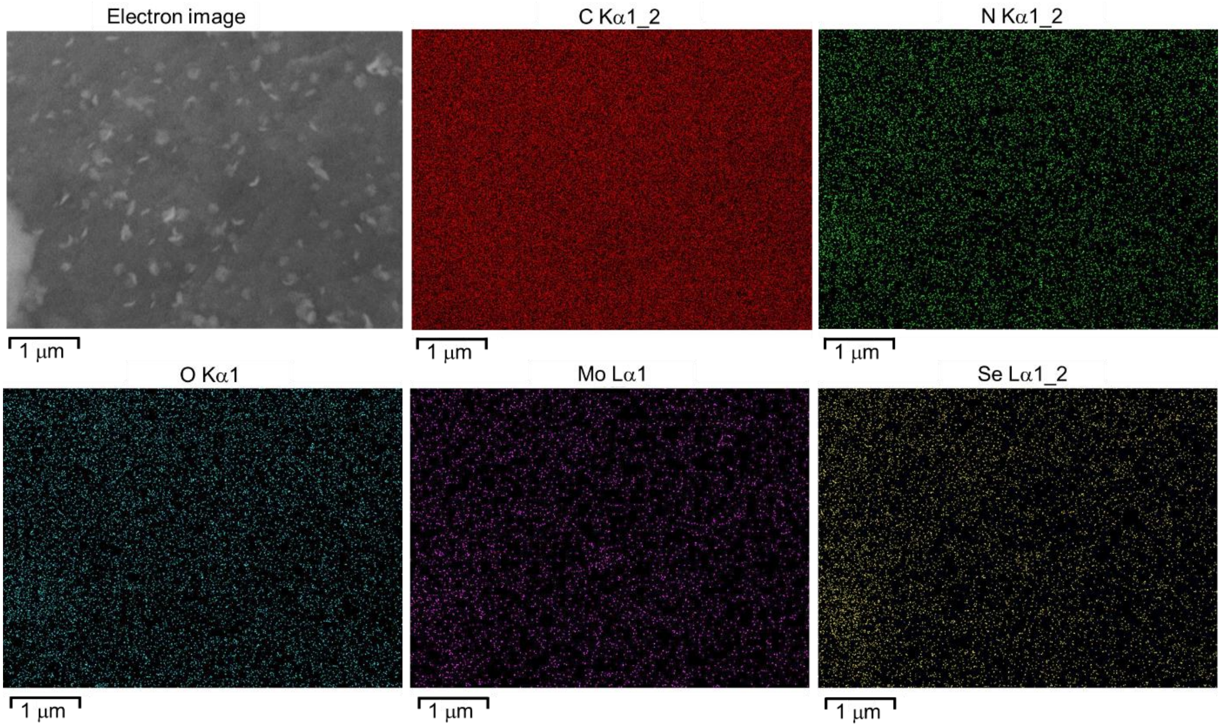

Figure S2 EDS mapping of $MoSe_2$/NGO material.

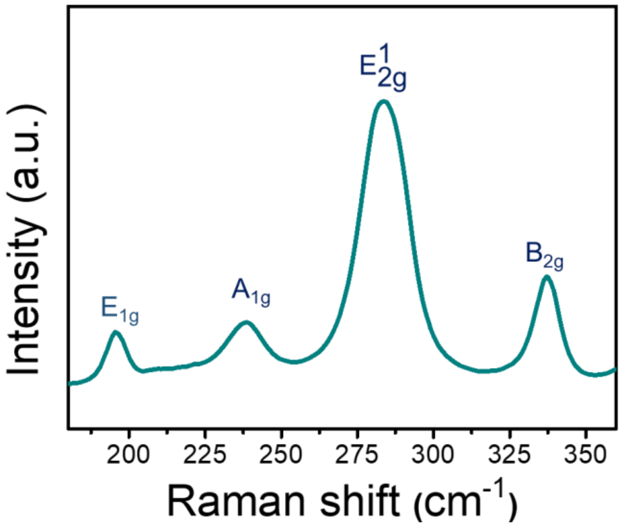

Figure S3 The magnification of Raman spectrum of the $MoSe_2$ sample in the low Raman shift region.

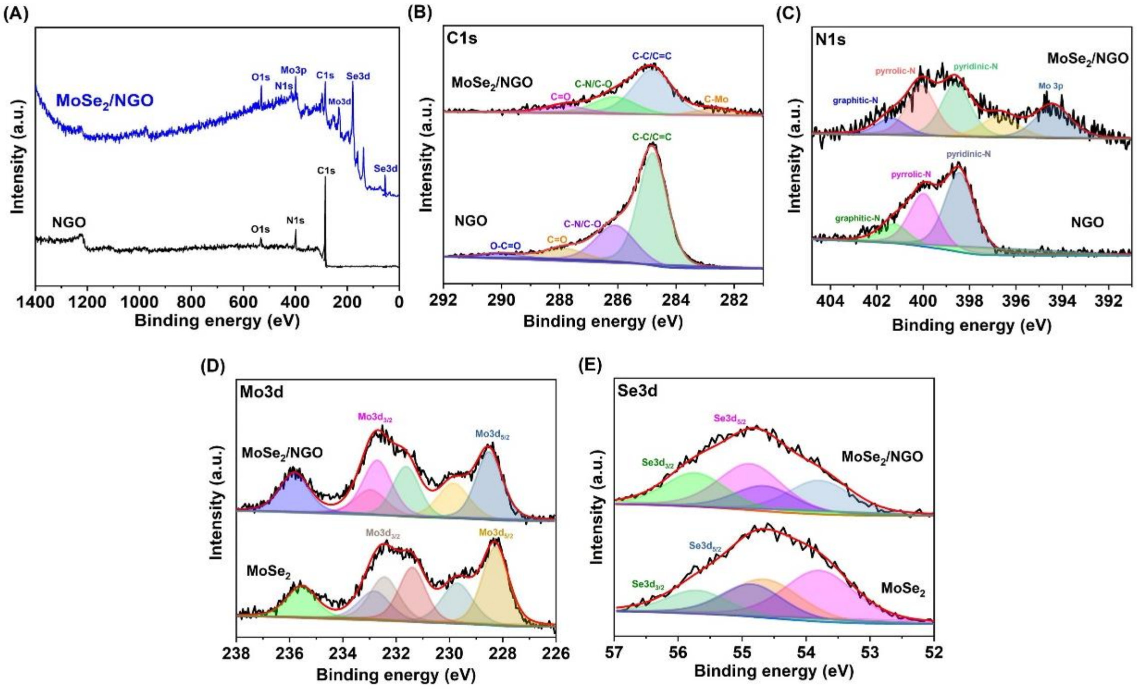

Figure S4 XPS survey spectra (A) and high-resolution XPS spectra in the C1s (B), N1s (C), Mo3d (D), and Se 3d (E) regions of NGO and $MoSe_2$/NGO.

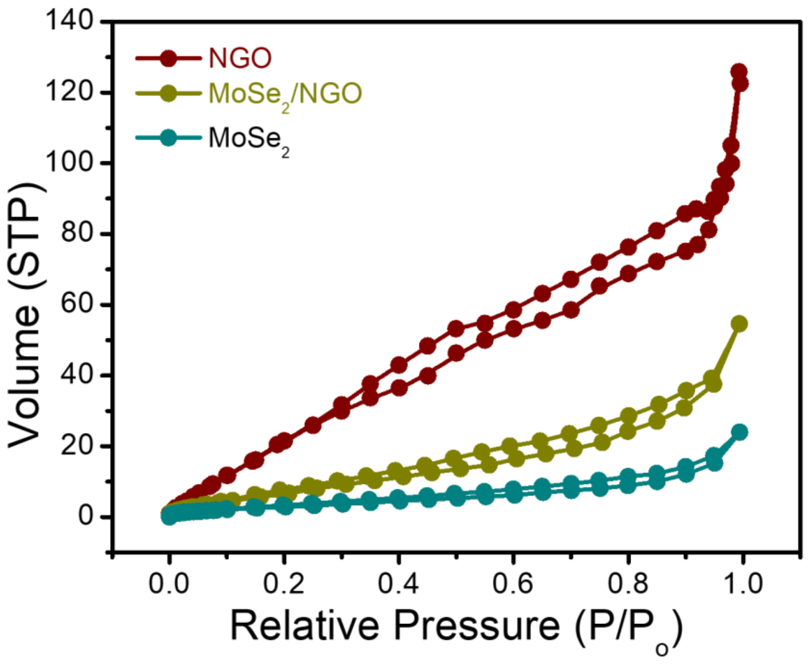

Figure S5 Nitrogen adsorption/desorption isotherm of $MoSe_2$, NGO and $MoSe_2$/NGO

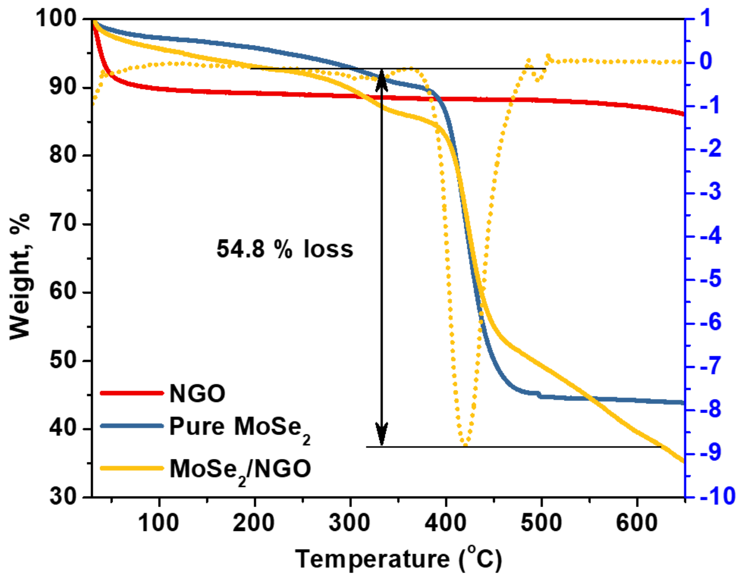

Figure S6 TGA and ΔTG curves of $MoSe_2$, NGO and $MoSe_2$/NGO

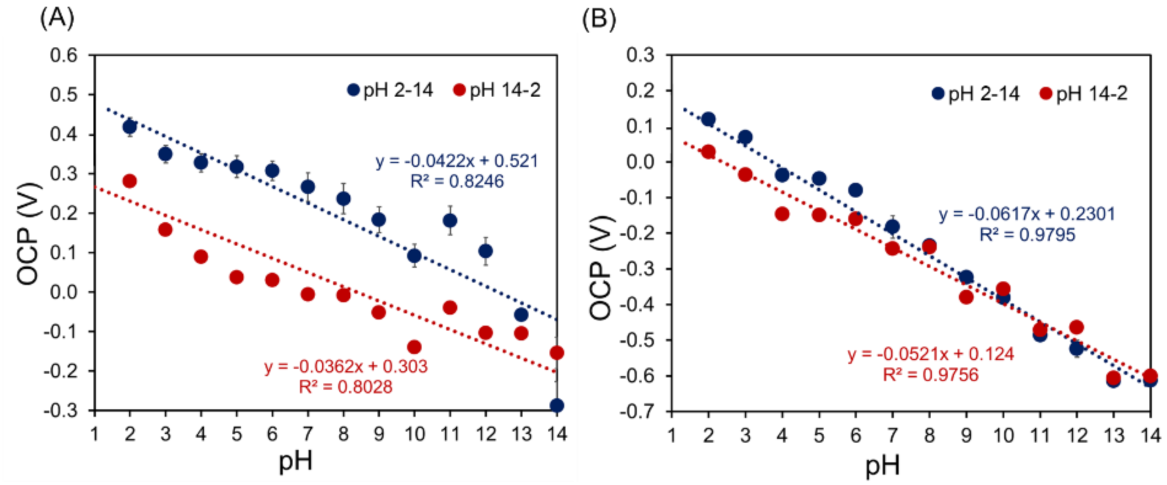

Figure S7 Calibration plot of open-circuit potential responses of different solution pH values from 2 to 14 (blue line) and 14 to 2 (red line) using NGO/SPE (A) and $MoSe_2$/SPE (B).

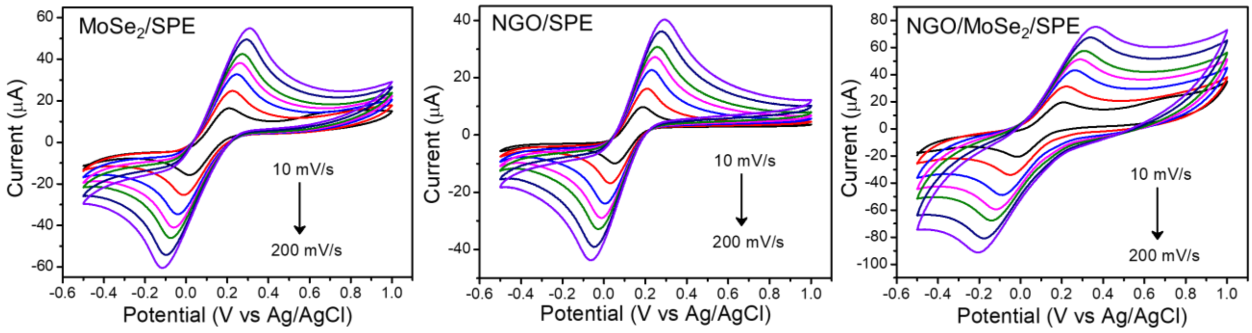

Figure S8 Cyclic voltammograms of for $MoSe_2$/SPE, NGO/SPE, and $MoSe_2$/NGO/SPE in a in 0.1 M KCl solution containing 10 mM $[Fe(CN)_6]^{4-/3-}$ at a scan rate of 10, 25, 50, 75, 100, 150 and 200 mV s^{-1}.

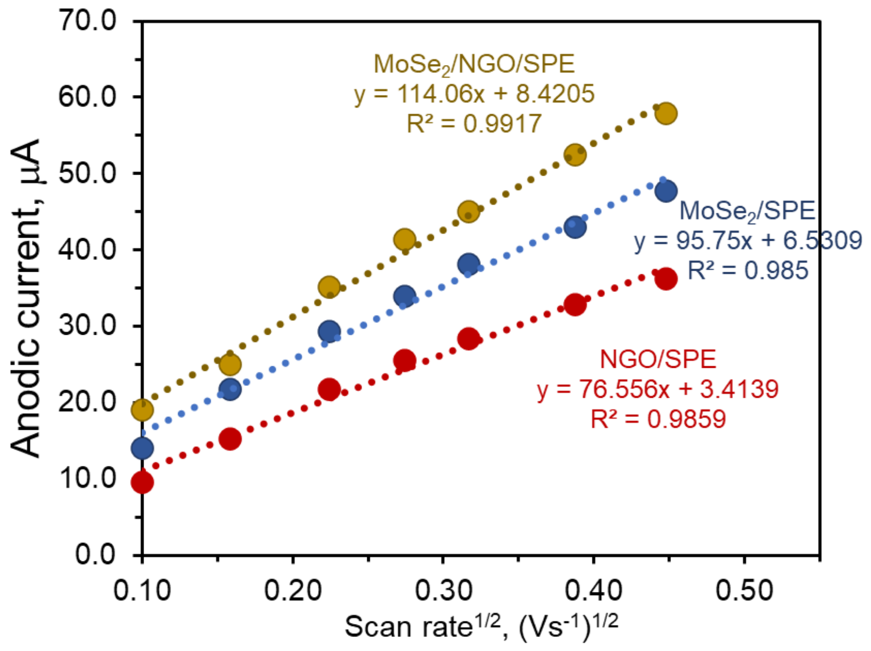

Figure S9 Anodic peak currents as a function of square root of scan rate for determining the effective surface areas of the $MoSe_2$/SPE, NGO/SPE, and $MoSe_2$/NGO/SPE

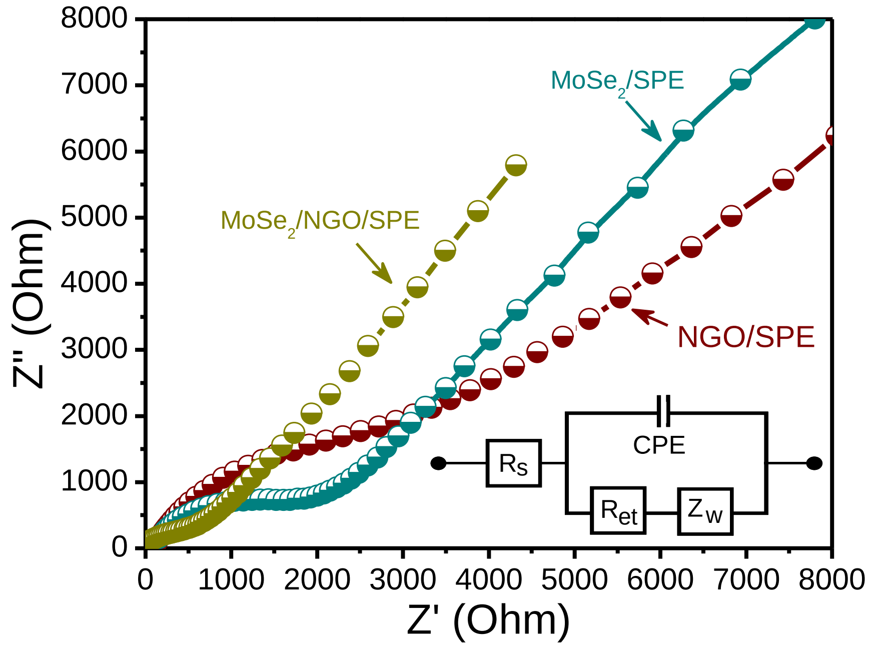

Figure S10 Impedance spectra (Nyquist plots) of 10 mM $[Fe(CN)_6]^{4-/3-}$ in 0.1 M KCl, for $MoSe_2$/SPE, NGO/SPE, and $MoSe_2$/NGO/SPE. The inset is the equivalent circuit used for the impedance data fitting; Rs is the solution resistance between working and reference electrodes, Zw is Warburg impedance; CPE is the double layer capacitance and Ret is the charge-transfer resistance.

Table S1 Calculated d-spacing and interlayer spacing of all the materials.

Materials	Position [2θ]	d-spacing [Å]	Crystal plane	Interlayer spacing (nm)
NGO	26.3<br>43.4	3.39<br>2.08	(002)<br>(100)	0.34<br>0.21
$MoSe_2$	9.0<br>33.0<br>54.3	9.82<br>2.71<br>1.69	(002)<br>(100)<br>(110)	0.98<br>0.27<br>0.17

Table S2 BET specific surface areas and average pore diameters of all materials**.**

Samples	BET surface area (m^2/g)	Average pore diameter (nm)
GO	297.12	1.91
NGO	436.86	2.99
$MoSe_2$	8.68	24.61
$MoSe_2$/NGO	13.01	21.17

Table S3 Comparison of performances of unmodified and modified SPE sensors.

Materials	pH 2-14		pH 14-2		Different sensitivity (mV)
	Sensitivity (mV/pH)	R^2	Sensitivity (mV/pH)	R^2	
NGO/SPE	42.2	0.8246	36.2	0.8028	6.0
$MoSe_2$/SPE	61.7	0.9795	52.1	0.9756	9.6
$MoSe_2$/NGO/SPE	61.3	0.9926	64.2	0.9945	2.9

References

1. J. N'Diaye, S. Poorahong, O. Hmam, G. C. Jiménez, R. Izquierdo and M. Siaj, *Membranes*, 2020, **10**.
